# T-Lymphocytes Enable Osteoblast Maturation via IL-17F during the Early Phase of Fracture Repair

**DOI:** 10.1371/journal.pone.0040044

**Published:** 2012-06-29

**Authors:** Diane Nam, Elaine Mau, Yufa Wang, David Wright, David Silkstone, Heather Whetstone, Cari Whyne, Benjamin Alman

**Affiliations:** 1 Division of Orthopaedic Surgery, Department of Surgery, Sunnybrook Research Institute, University of Toronto, Toronto, Ontario, Canada; 2 Department of Developmental Biology, The Hospital for Sick Children, University of Toronto, Toronto, Ontario, Canada; University Hospital Jena, Germany

## Abstract

While it is well known that the presence of lymphocytes and cytokines are important for fracture healing, the exact role of the various cytokines expressed by cells of the immune system on osteoblast biology remains unclear. To study the role of inflammatory cytokines in fracture repair, we studied tibial bone healing in wild-type and *Rag1^−/−^* mice. Histological analysis, µCT stereology, biomechanical testing, calcein staining and quantitative RNA gene expression studies were performed on healing tibial fractures. These data provide support for *Rag1^−/−^* mice as a model of impaired fracture healing compared to wild-type. Moreover, the pro-inflammatory cytokine, IL-17F, was found to be a key mediator in the cellular response of the immune system in osteogenesis. *In vitro* studies showed that IL-17F alone stimulated osteoblast maturation. We propose a model in which the Th17 subset of T-lymphocytes produces IL-17F to stimulate bone healing. This is a pivotal link in advancing our current understanding of the molecular and cellular basis of fracture healing, which in turn may aid in optimizing fracture management and in the treatment of impaired bone healing.

## Introduction

The molecular and cellular regulation of fracture healing is not completely understood, yet such knowledge is critical to developing treatments to optimize bone repair and remodeling. There is growing evidence that inflammation plays a crucial role in early fracture repair [1]–[5]. In mouse models, interleukin (IL) −1, −6 and tumor necrosis factor α (TNFα) expression is present in the fracture site within the first 24 h period post-injury with both TNFα and IL-6 knockout mice demonstrating delayed endochondral repair and callus remodeling [3], [6]. Similar to other injury states, such as wound healing [7], the immunologic response is pivotal in initiating the necessary triggers that result in cellular differentiation required for successful bone healing. Although most osteoimmunology research has centered on the areas of inflammatory and metabolic bone processes [8], in diseases such as rheumatoid arthritis and osteoporosis, interest within the context of other pathological conditions, such as fracture healing, has been a more recent focus. The molecular links between bone and the immune system have emerged from the identification of receptor activator of nuclear factor-kappaB/ligand (RANK) and RANKL as key osteoclastogenic molecules. However, immunological regulation of the osteoblast has been a particularly poorly understood topic to date. While it is well known that there exists a mutual interaction between osteoblast and osteoclast cells through RANK-RANKL signaling [8]–[12], the role of lymphocytes and cytokines in osteoblast biology with respect to osteoblast activation and maturation during fracture healing remains unknown [13]–[15].

In normal fracture healing, osteoblasts synthesize osteoid matrix which is eventually mineralized to produce bone. Cells of the osteoblast lineage include bone-lining cells and osteocytes, the latter of which becomes embedded in the lacunae as the surrounding bone is formed. Runt-related transcription factor 2/core binding factor 1 (*Runx2*) and Osterix (*OSx*) are both known transcription factors essential for early osteoblast differentiation. Similarly, activation of the canonical Wnt and bone morphogenic protein (BMP) signaling pathways are known to play a role in osteoblast differentiation [16]. Yet exactly how these pathways are affected by the immune system remains an ongoing area of investigation.

Mice lacking recombinase activating genes *Rag1 or Rag2* are unable to form T-cell or B-cell receptors and hence, completely lack mature T and B lymphocytes [17], [18]. The *Rag1* and *Rag2* proteins act in combination as a heterodimer to facilitate the rearrangement of variable (V), diversity (D) and joining (J) genes required for the generation of immunoglobulin and T-cell receptors. This rearrangement is necessary for diversity in antigen recognition and is permissive in allowing the developing B-cells and T-cells to mature and enter the circulation. In turn, *Rag1* deficient mice are devoid of any lymphocytic sources of interleukins and provide a preclinical model of fracture healing in the absence of these secreted factors. Thus, the *Rag1^−/−^* mouse is a useful animal to elucidate the physiological role of T-cells and their subtypes on osteoblast differentiation during fracture healing.

## Materials and Methods

### Ethics Statement

All studies followed the Canadian Council of Animal Care (CCAC) guidelines and all procedures were approved by Sunnybrook Health Sciences Centre Animal Care Committee. (AUP#09-407). All surgery was performed under isoflurane gas anesthesia, and all efforts were made to minimize suffering.

### Mice

B6.129S7-*Rag1^tm1Mom^* ^/J^ (*Rag1^−/−^*) and C7BL/6J wildtype (WT) male 12-week old mice were used (Jackson Laboratory, Bar Harbor, Maine). A longitudinal incision was made over the knee, and a 0.5 mm hole was made just proximal to the tibial tubercle and lateral to the patellar tendon. The tibia was pre-stabilized by placing an 0.9 mm intramedullary pin (Fine Science Tools, http://www.finescience.com/) in the marrow space as previously reported [19] with the following modifications. The fracture was generated by an open osteotomy in the mid shaft of the tibia through a separate small anterolateral incision with minimal soft tissue dissection. Previous data show that a fracture generated in this manner heals through both endochondral and intramembranous ossification [20]. It allows for consistency in fracture generation with respect to fracture level, orientation (ie. transverse vs. multi-fragmentary) and controlling for the fibula to remain intact, thus minimizing for variability between specimens and allowing a more homogeneous assessment of fracture callus cell types. The animals were allowed unrestricted weight-bearing immediately following surgery. Mice were euthanized at different time points post fracture and the limbs were harvested for analysis.

### Cytokine/Chemokine Measurements

The serums of WT and *Rag1^−/−^* mice were drawn at 2 days post-fracture for detection and quantification of immunologic proteins in the early phase of repair. The levels of 32 cytokines/chemokines (Eotaxin, G-CSF, GM-CSF, IFN-γ, IL-1α, IL-1β, IL-2, IL-3, IL-4, IL-5, IL-6, IL-7, IL-9, IL-10, IL-12 (p40), IL-12 (p70), IL-13, IL-15, IL-17, IP-10, KC, LIF, LIX, MCP-1, M-CSF, MIG, MIP-1α, MIP-1β, MIP-2, RANTES, TNFα, VEGF) were assessed with the Mouse Cytokine/Chemokine MilliplexTM Map kit (Millipore, Billerica, MA) using Luminex® technology according to the manufacturer’s instructions and assayed with the Luminex100ISTM system by Linco Research, Inc. The cytokine detection limit for this assay was 3.2 pg/mL.

### *In vitro* Studies

To determine whether the presence or absence of mature T-cells influence osteoblast differentiation, primary mesenchymal stromal cells (MSC) were harvested from bilateral femurs, tibias and humeri of 12 week old WT and *Rag1^−/−^* mice. After lysis of red blood cells using ACK lysis buffer, 5.0×10^6^/mL cells were seeded per 12 multi-well plate for 7 days in a 37°C incubator (Becton Dickinson) in αMEM (Wisent, St-Bruno, Quebec) containing high glucose supplemented with 100 U/ml penicillin, 100 µg/ml streptomycin, and 10% fetal calf serum. At day 4, half of the media containing non-adherent cells was exchanged with fresh media and at day 7, the media was changed completely to osteoblast differentiation media (αMEM supplemented with 50 µg/ml ascorbic acid (Sigma-Aldrich, St. Louis, MO), 10*^−^* ^8^ M dexamethasone (Sigma-Aldrich, St. Louis, MO), and 8 mM β-glycerophosphate (Sigma-Aldrich, St. Louis, MO). Cells were harvested at 20 days for RNA extraction and analysis with RNeasy® Plus Mini Kit (Qiagen, Valencia, CA) for quantitative real-time RT-PCR and staining with Alizarin red.

Murine pre-osteoblast cell line, MC3T3-E1, were maintained in αMEM supplemented with 2.5% fetal bovine serum (FBS; Gibco, Invitrogen) and 100 U/ml penicillin and 100 µg/ml streptomycin 37°C in 5% CO_2_ atmosphere. The spontaneous differentiation into osteoblasts of MC3T3-E1 and primary mesenchymal stromal cells was induced by osteoblast differentiation media as above. For IL-17F treatment, 2.5×10^5^ MC3T3-E1 cells and 2.5×10^5^ primary mesenchymal stromal cells as prepared above were seeded in 6-well plates with αMEM added with 20 ng/ml of IL17F (R&D systems, Minneapolis, MN) for 4 days; similarly for TGFβ treatment, 10 ng/ml (R&D systems, Minneapolis, MN) was used. Cells were harvested and RNA was extracted for analysis with osteoblast and cytokine markers (Table 1).

**Table 1:** List of Primers.

No.	Gene		Sequence (5′ to 3′′)
1	GAPDH	Forward	AACTTTGGCATTGTGGAAGG
		Reverse	ACACATTGGGGGTAGGAACA
2	IFNγ	Forward	ACTGGCAAAAGGATGGTGAC
		Reverse	TGAGCTCATTGAATGCTTGG
3	TNFα	Forward	AGCCCCCAGTCTGTATCCTT
		Reverse	CTCCCTTTGCAGAACTCAGG
4	TGFβ	Forward	TTGCTTCAGCTCCACAGAGA
		Reverse	TGGTTGTAGAGGGCAAGGAC
5	IL-1	Forward	CCCGTCCTTAAAGCTGTCTG
		Reverse	AATTGGAATCCAGGGGAAAC
6	IL-4	Forward	TCAACCCCCAGCTAGTTGTC
		Reverse	TGTTCTTCGTTGCTGTGAGG
7	IL-6	Forward	AGTTGCCTTCTTGGGACTGA
		Reverse	TCCACGATTTCCCAGAGAAC
8	IL-10	Forward	CCAAGCCTTATCGGAAATGA
		Reverse	TTTTCACAGGGGAGAAATCG
9	IL-12b	Forward	AGGTGCGTTCCTCGTAGAGA
		Reverse	AAAGCCAACCAAGCAGAAGA
10	IL-13	Forward	CAGCTCCCTGGTTCTCTCAC
		Reverse	CCACACTCCATACCATGCTG
11	IL-17A	Forward	TCCAGAAGGCCCTCAGACTA
		Reverse	AGCATCTTCTCGACCCTGAA
12	IL-17F	Forward	GTGTTCCCAATGCCTCACTT
		Reverse	GTGCTTCTTCCTTGCCAGTC
13	IL-23	Forward	GACTCAGCCAACTCCTCCAG
		Reverse	GGCACTAAGGGCTCAGTCAG
14	ALP	Forward	CCAGCAGGTTTCTCTCTTGG
		Reverse	CTGGGAGTCTCATCCTGAGC
15	Col1	Forward	GAGCGGAGAGTACTGGATCG
		Reverse	GCTTCTTTTCCTTGGGGTTC
16	Col2	Forward	GCCAAGACCTGAAACTCTGC
		Reverse	GCCATAGCTGAAGTGGAAGC
17	BSP2	Forward	AAAGTGAAGGAAAGCGACGA
		Reverse	GTTCCTTCTGCACCTGCTTC
18	Runx2	Forward	CCCAGCCACCTTTACCTACA
		Reverse	TATGGAGTGCTGCTGGTCTG
19	Osteocalcin	Forward	CTTGGTGCACACCTAGCAGA
		Reverse	ACCTTATTGCCCTCCTGCTT

### Fracture Analysis

Tibias were harvested at 3, 7, 14, 21, 28 and 35 days post-fracture. Tibias harvested at 3 and 7 days were fixed in 4% paraformaldehyde for histological analysis, decalcified in 10% EDTA (pH 7.4) for 3 weeks, dehydrated and embedded in paraffin. Sections 10 µm thick were prepared with Safranin O staining (Sigma) (n = 3) for histologic analysis. Immunohistochemistry (IHC) was performed using ABC Kit (Vector Laboratories, Burlingame, CA) following the standard manufacturer protocol. The monoclonal anti-CD3 with goat anti-rabbit and anti-mouse immunoglobulin (Ig) from Abcam (Cambridge, CA) was used. The anti-mouse IL-17F antibody from R&D Systems (Minneapolis, MN) was used. Tibias harvested at 3 and 7 days also underwent total RNA isolation from their callus. The callus was snap frozen in liquid nitrogen, processed by BioPulvenizer (MidSci) and total RNA was extracted using TRIZOL (Invitrogen). Quantitative real-time reverse transcriptase polymerase chain reaction (RT-PCR) was performed with StepOnePlus system (Applied Biosystems) using SYBR Green (Bio-Rad). The primers are listed in Table 1.

Fractured and unfractured tibias harvested at 14, 21 and 28 days for both WT and *Rag1^−/−^* mice were fluorochrome labeled with calcein green as described by van Gaalen et al, 2010 [21] (n = 3 per group). At 2 and 9 days prior to harvest, mice were given 30 mg/kg calcein green (Sigma-Aldrich, St. Louis, MO) via peritoneal injections to sequentially label new bone deposition and bone remodeling during a one week healing period. Briefly, harvested tibias were fixed in 70% ethanol. In vacuum jars, specimens were sequentially dehydrated using ascending acetone concentrations. Following dehydration, the infiltration process was performed with increasing concentrations of Spurr resin (50, 80, 2×100%) (SPI-Pon™ 812 Embedding Resin System). Sections of 7 µm were cut in the long axis of the tibia using a rotary microtome (Leica RM 2165) for fluorescent microscopy imaging (495 nm/521 nm, FITC) and analysis (Bioquant, Nashville, TN).

### Micro-computed Tomography Analysis

WT and *Rag1^−/−^* mice tibias harvested at 28 and 35 days were used for micro-computed tomography (µCT) based stereologic analysis and standard torsional biomechanical testing. Characterization of callus geometry and mineralization was analyzed using high resolution µCT. Samples were scanned at an isotropic voxel size of 8 µm (SkyScan 1172, SkyScan, Belgium) using a voltage of 50 kV, a current of 160 µA, and a 0.5 mm aluminum filter. Samples were placed with the long axis of the tibia coincident with the vertical axis of the scanner, and scanned in combination with calibrated bone density phantoms to allow direct measurements of bone density from the scans using a linear relationship.

Three-dimensional callus properties of geometry and mineralization were calculated using the custom processing option available in CTAn software (SkyScan, Belgium) and in-house code in AmiraDEV (VGS, Germany). Structural and material parameters were calculated by first segmenting the images with a global threshold value of 0.2 gHA/cm^3^ to define voxels corresponding to bone [22]. Following a despeckling filter to remove noise in the images, a region of interest (ROI) was fitted around the callus on subsequent axial slices using an adaptive contouring algorithm which ignored holes in the outside of the callus of less than 50 pixels. Measurements of the callus properties in the ROI included Bone Volume (BV, mm^3^), Total Callus Volume (TV, mm^3^), Bone Volume Fraction (BV/TV, %), Trabecular Thickness (Tb.Th., mm), Trabecular Number (Tb.N., 1/mm), Trabecular Separation (Tb.Sp., mm), Mean Bone Mineral Density (BMD, gHA/cm^3^), Mean Tissue Mineral Density (TMD, gHA/cm^3^) and Torsional Rigidity (CTRA, kNmm^2^) [23].

### Mechanical Testing

Torsional strength and torsional stiffness were measured at 28 and 35 days using a MTS Bionix 858 (MTS Systems, MN, USA) materials testing system. Prior to testing, intramedullary pins and fibulas were removed from all tibias using sharp bone scissors. Each tibia was then aligned longitudinally to the loading axis of the MTS and potted proximal and distal to the fracture callus in polymethylmethacrylate. The gauge length, defined as the length between the two potting casings, was kept consistent for each sample. Torque was measured during the application of angular displacement (1°/second) until failure or to a maximum displacement of 30°. The maximum torque, twist angle at failure, and torsional stiffness were calculated based on the generated load displacement data. Torsional strength was defined as the maximum load sustained during loading, and the torsional stiffness was defined as the slope of the line extending to the point of maximum sustained torque.

### Statistical Analysis

Data were expressed as mean ± standard deviation. Statistical differences were calculated using a Student *t* test. Unless stated otherwise, 10 animals were utilized per group for the RT-PCR expression data; 9 animals (WT) and 8 animals (*Rag1^−/−^*) were used for µCT stereology and biomechanical analyses. A *p* value below 0.05 was considered statistically significant.

## Results

### T-cells are Present in the Early Phase of Fracture Repair and Correlates to Bone Marker Gene Expression

**Figure 1:**
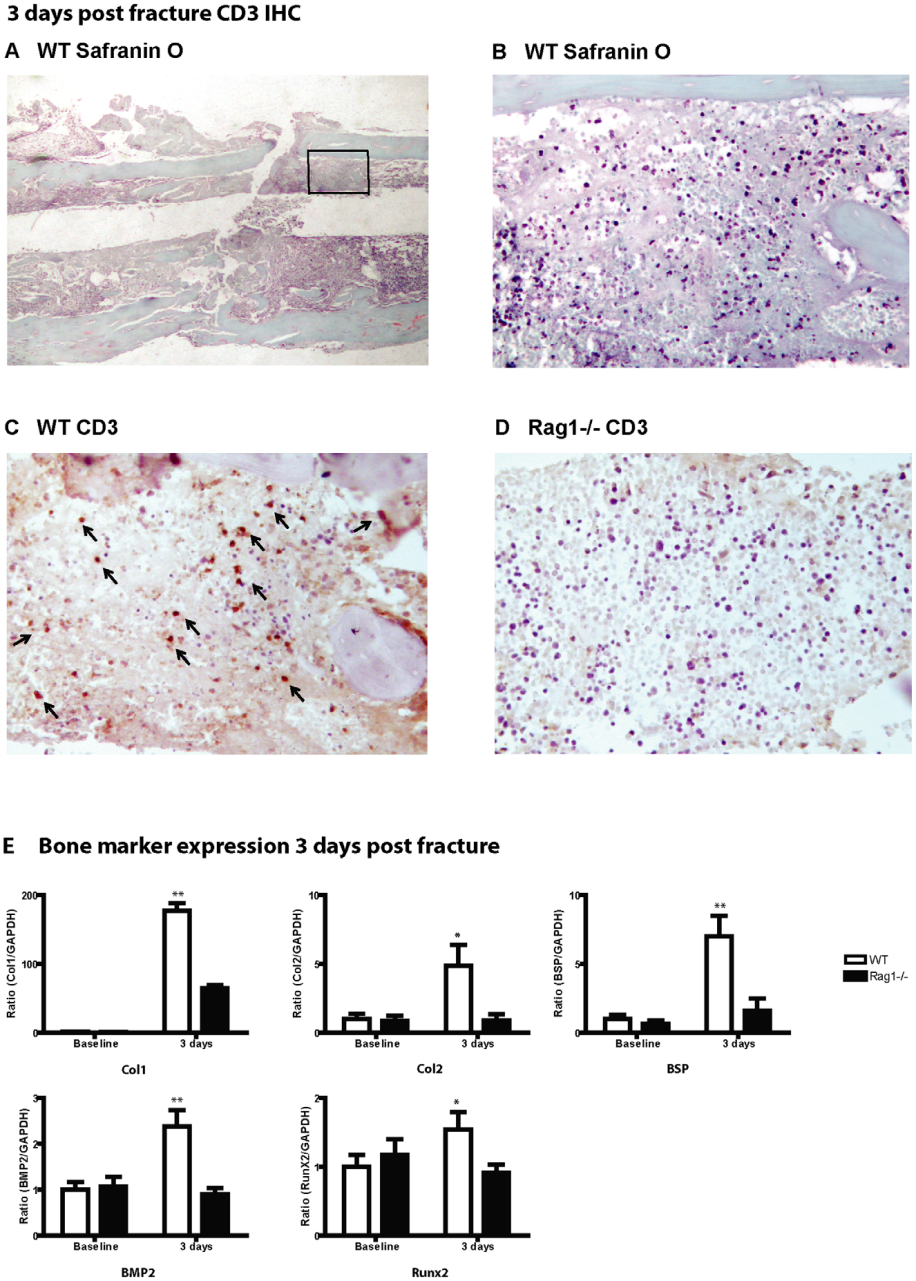
T-cell presence and increased bone marker expression are demonstrated in early fracture repair. (**A**) Although no cartilage or new bone formation is expected at 3 days post tibial fracture, low magnification (20X) Safranin O staining of WT mice at this time point helps to illustrate the region of interest (inset). (**B**) Higher magnification images corroborate the histology in this region and (**C**) positive CD3 staining of T-cells localizing to the endosteal fracture hematoma in contiguous sections (200X). Immunohistochemistry analysis confirms presence of T-cell infiltration (CD3 – black arrows) in WT mice. (**D**) In a similar region, no positive staining was seen in *Rag1^−/−^* mice (200X). (**E**) T-cell presence is shown to correlate with gene expression of mature bone markers detected on quantitative RT-PCR normalized to GAPDH. Expression of Col1 (p<0.01), Col2 (p<0.05), BSP (p<0.01), BMP2 (p<0.01) and Runx2 (p<0.05) were increased in WT compared to *Rag1^−/−^* mice at 3 days post fracture.

To confirm the role of T-cells as an early responder in the fracture healing cascade, immunohistochemistry of WT mice fracture callus was analyzed. CD3, a marker ubiquitously expressed in T-cells, was used to identify these cells in the fracture region. A low magnification Safranin O section (Fig. 1A) was used to illustrate the region of interest whereby higher magnification images corroborated the histology (Fig. 1B) and positive CD3 staining of T-cells which characteristically localized adjacent to the endosteum in the fracture hematoma at 3 days post fracture in all sections (Fig. 1C). The *Rag1^−/−^* mice at 3 days post fracture did not show any specific T-cell staining in multiple high power field regions in the fracture callus (Fig. 1D). The distribution of T-cells predominantly in the endosteal fracture hematoma was representative in the all the WT mice CD3 immunohistochemistry during this early phase of healing. Following the inflammatory phase at 7 days post fracture, immunostaining results showed a reduction in the presence of T-cells at the fracture site in similar sections during the later stages of bone healing (data not shown). Although the presence of B-cells in the fracture hematoma has been observed [12], antigen processing and clonal expansion is not a known early response of fracture healing. While CD45R antibody staining is commonly used as a marker of B-cells, there are reported examples of cross-reactivity in the mouse with positive immunofluorescence and signal transduction of T-cells using CD45R [24] and therefore, any positive CD45R staining would be equivocal in concluding B-cell specificity.

Interestingly, while the relative absence of T-cells in the later phases of fracture healing (7, 14, 28 days post fracture) showed no significant differences in mature bone marker expression between WT and *Rag1^−/−^* mice (data not shown), the presence of T-cells at 3 days post fracture correlated with the level of expression of mature bone markers, detected using real-time quantitative RT-PCR normalized to GAPDH. Expression of Collagen 1 (Col1) (p<0.01), Collagen 2 (Col2) (p<0.05), bone sialoprotein (BSP) (p<0.01), BMP2 (p<0.01) and Runx2 (p<0.05) were significantly increased in WT compared to *Rag1^−/−^* mice at 3 days post fracture (Fig. 1E). The *Rag1^−/−^* mice which lack mature T-cells generally showed a failure to increase bone marker expression 3 days post fracture. Both Col1 and BSP were more significantly decreased at a *p value* <0.01. BSP has the biophysical and chemical properties of a nucleator and its temporo-spatial expression has shown to coincide with de novo mineralization in bone [25]. The significant decrease in BSP suggests an impairment in *Rag1^−/−^* mice in its ability to up-regulate BSP in osteoblasts to form bone.

### Pro-inflammatory Cytokine Expression is Decreased and Anti-inflammatory Cytokine Expression is Increased in the Fracture Callus of *Rag1^−/−^* Mice

**Figure 2:**
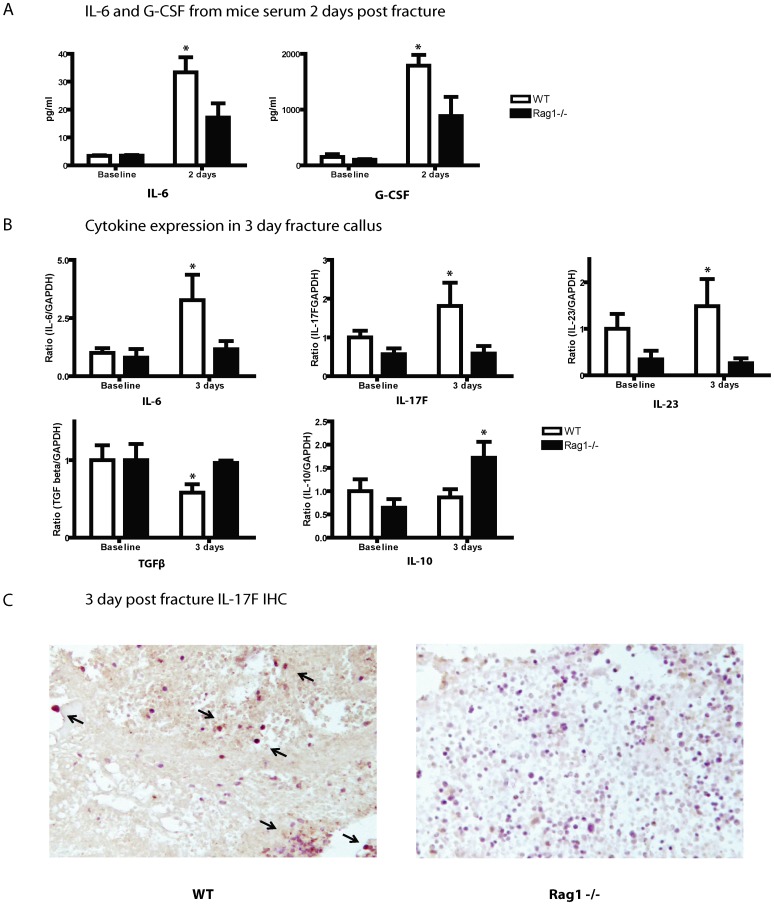
Cytokine expression during the early phase of fracture repair. (**A**) WT and *Rag1^−/−^* mice serums drawn 2 days post-fracture show elevated IL-6 and G-CSF levels compared to unfractured baseline mice. However, WT mice were found to have twice the increase in cytokine levels of IL-6 and G-CSF compared to *Rag1^−/−^* mice during this early phase of fracture healing (p<0.05). (**B**) Fracture callus RNA expression analysis of cytokine levels at a similar early time point post fracture, shows a greater than 2 fold up-regulation of pro-inflammatory cytokines IL-6, IL-17F and IL-23 in WT mice compared to *Rag1^−/−^* and baseline mice (p<0.05). Conversely, expression of anti-inflammatory cytokine IL-10 is up-regulated in *Rag1^−/−^* and TGFβ is down-regulated in WT mice. (**C**) Immunohistochemistry analysis during the early phase of fracture healing at 3 days post fracture confirmed IL-17F positive staining (black arrows) in WT mice similar to the distribution of CD3 staining shown previously with no positive IL-17F staining in *Rag1^−/−^* mice (200X).

To gain further insight as to the relevant cytokines involved during early T-cell activation and osteoblast maturation, thirty-two cytokines and chemokines were profiled and their expression analyzed using a protein assay of mice serum 2 days post fracture. Cytokines IL-6 and granulocyte colony-stimulating factor (G-CSF) were significantly lower in *Rag1^−/−^* mice compared to WT mice (p<0.05) (Fig. 2A). As well, eotaxin (p<0.05), IL-12 (p40) (p<0.05) and CXC motif chemokine 10 (IP-10) (p<0.05) were elevated in *Rag1^−/−^* mice compared to WT whereas CXC chemokine ligand 5 (LIX) (p<0.01) and CXC motif ligand 9 (MIG) (p<0.01) were decreased in *Rag1^−/−^* mice (data not shown). The remainder of the cytokine/chemokine panel showed no significant difference in expression between WT and *Rag1^−/−^* mice at this 2 day time point (n = 3 per group).

The systemic changes in cytokine and chemokine expression during the early phase of healing were further assessed locally at the fracture callus. Quantitative RT-PCR of the extracted RNA at 3, 7, 14 and 28 days was screened using several known cytokines previously identified in fracture healing (Table 1). The regulatory patterns of the cytokines at 3 days showed a characteristic grouping. The pro-inflammatory cytokines IL-6 (p<0.05), IL-17F (p<0.05) and IL-23 (p<0.05) were expressed at significantly lower levels in the *Rag1^−/−^* mice compared to WT, while the anti-inflammatory cytokines IL-10 (p<0.05) and TGFβ (p<0.05) were expressed at much higher levels relative to WT mice (Fig. 2B). Of note, IL-17A which has an important role in regulating osteoclastogenesis, was found to be undetectable in WT and *Rag1^−/−^* mice at any of the early or late time points studied indicating that IL-17A is not induced during the fracture repair process. Immunohistochemistry was completed using an IL-17F antibody to confirm the presence of a pro-inflammatory cytokine in the fracture site during the early phase of healing in WT and *Rag1^−/−^* mice 3 days post fracture (Fig. 2C). The presence of IL-17F was noted in the WT mice fracture hematoma similar to the distribution of the previous CD3 staining of T-cells. Predictably, the *Rag1^−/−^* mice showed no appreciable IL-17F staining in this same region. Unfractured and 7 day post fractured tibias demonstrated no appreciable positive IL-17F staining (data not shown). However, the level of TGFβ expression in *Rag1^−/−^* mice was comparable to baseline, unbroken limbs suggesting a constitutive activity of TGFβ in WT mice that is down-regulated post fracture. The TGFβ down-regulation in *Rag1^−/−^* mice was not observed. Similarly, the up-regulation of IL-6 and IL-17F in *Rag1^−/−^* mice was also absent. In fact, IL-10 was the only cytokine up-regulated post fracture in the *Rag1^−/−^* mice.

At 7, 14 and 28 days, no significant differences were seen between the *Rag1^−/−^* and WT mice in their cytokine expression, except IL-6, which still remained decreased in *Rag1^−/−^* compared to WT mice (data not shown). Thus, the observed regulatory grouping of cytokines present at 3 days was only specific to the early phase of fracture healing.

### Pro-inflammatory Cytokine, IL-17F, Stimulates Bone Maturation

**Figure 3:**
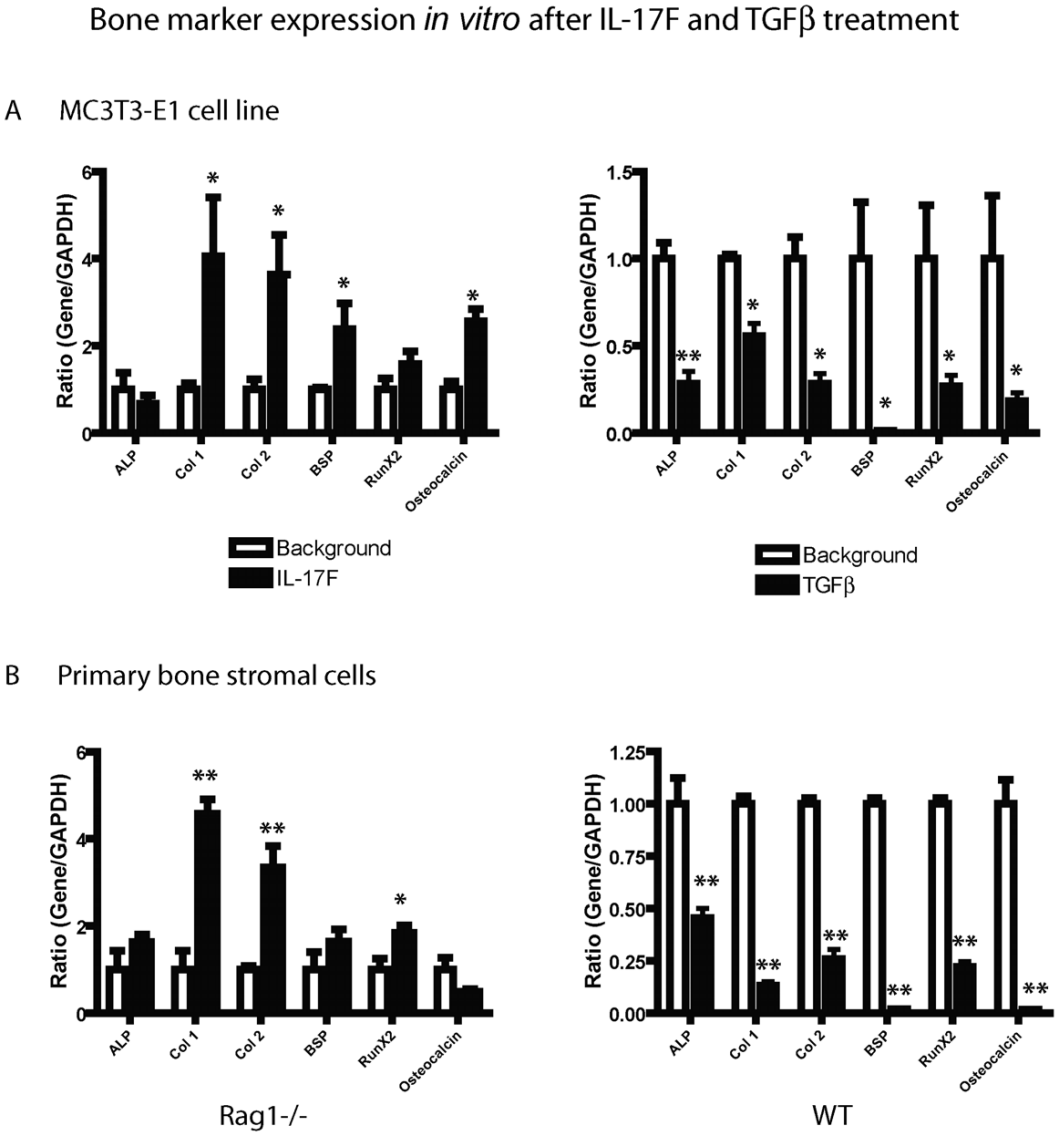
IL-17F promotes osteoblast maturation. (**A**) Treatment of MC3T3-E1 pre-osteoblast cell line cultures directly with pro-inflammatory cytokine IL-17F showed increased bone marker gene expression of Col1, Col2, BSP and osteocalcin, whereas, anti-inflammatory cytokine TGFβ treatment inhibited osteoblast maturation and showed significant decreases in expression of the entire panel of bone markers analyzed. (**B**) Left: Treatment of *Rag1^−/−^* mice primary mesenchymal stromal cell cultures directly with pro-inflammatory cytokine IL-17F showed increased bone marker gene expression of Col1, Col2, and Runx2. Right: In contrast, treatment with anti-inflammatory cytokine TGFβ inhibited WT mice primary mesenchymal stromal cell maturation and showed significant decreases in expression of all the bone markers analyzed. (*p<0.05 and **p<0.01).

To examine the effects of these cytokines on the osteoblast directly, a MC3T3-E1 pre-osteoblastic cell line was cultured and treated with IL-17F or TGFβ. Cells treated with the pro-inflammatory cytokine, IL-17F (Fig. 3A left) showed significantly increased expression of mature bone markers Col1 (p<0.05), Col2 (p<0.05), BSP (p<0.05) and osteocalcin (p<0.05) compared to untreated cells. Alkaline phosphatase (ALP), a surrogate marker of early osteogenic differentiation and Runx2, an upstream transcription factor for proteins of osteoblast formation showed no significant increase with IL-17F treatment. Cells treated with TGFβ (Fig. 3A right) exhibited a reciprocal effect with significantly decreased levels of all bone markers ALP (p<0.01), Col1 (p<0.05, Col2 (p<0.05), BSP (p<0.05), Runx2 (p<0.05) and osteocalcin (p<0.05). This confirmed an inhibitory effect on osteoblast maturation in these pre-osteoblastic cells with TGFβ treatment.

To determine the effects of IL-17F and TGFβ on *Rag1^−/−^* and WT mice, primary mesenchymal stromal cell cultures were studied. *Rag1^−/−^* mice mesenchymal stromal cells treated with IL-17F promoted osteoblast maturation and showed increased bone marker expression of Col 1, Col2 and Runx2 (Fig. 3B left). Primary mesenchymal stromal cells from WT mice treated with TGFβ, suppressed osteoblast maturation and showed a significant decrease in the expression of all the bone markers assessed (Fig. 3B right).

### There is a Reduction in Osteogenesis during Fracture Repair in *Rag1^−/−^* Mice

**Figure 4:**
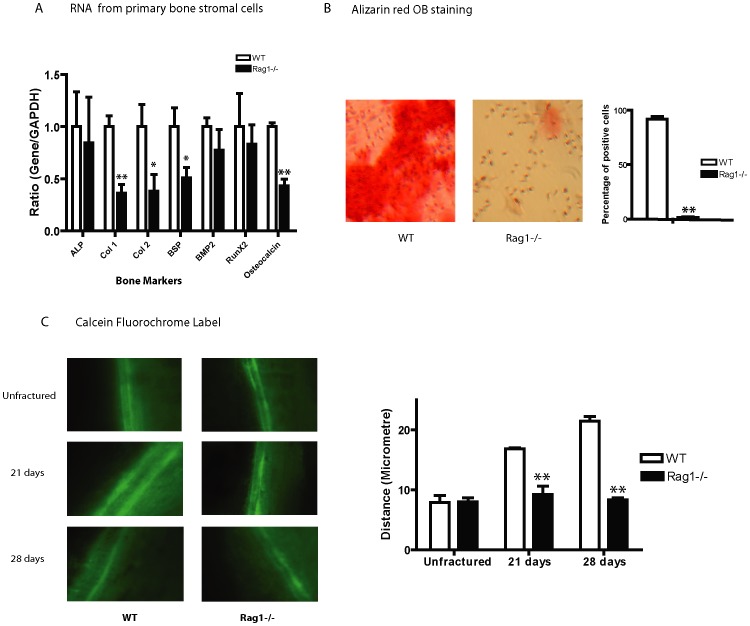
Osteogenesis is reduced in *Rag1* *^−^* ^***/****−*^ **mice during fracture repair.** (**A**) *In vitro* cultures of primary mesenchymal stromal cells differentiated to osteoblast colony-forming units (CFU) showed lower gene expression levels of bone markers Col1, Col2 and BSP and osteocalcin in *Rag1^−/−^* mice compared to WT. *p<0.05, **p<0.01 (n = 10). (**B**) Alizarin red staining of osteoblast CFU showed significant decrease in mineralization at 20 days of culture in *Rag1^−/−^* compared to WT. CFU-osteoblast was quantified by direct counting of all stained nodules positive to Alizarin Red using light microscopy. (**C**) Digital fluorescent microscopy images of calcein green administered mice 2 and 9 days prior to harvest at 21 and 28 days post fracture exhibited a smaller distance measured between mineralization fronts and hence, less bone formation in *Rag1^−/−^* compared to WT mice (p<0.05). Unfractured limbs showed no significant differences in bone formation.

*In vitro* cultures of primary mesenchymal stromal cells treated with osteoblast differentiation media were performed on unfractured WT and *Rag1^−/−^* mice to assess for any inherent differences in mineralization potential. RNA extracted from *Rag1^−/−^* mice primary bone cultures (Fig. 4A) demonstrated decreased expression of mature bone markers Col1 (p<0.01), Col2 (p<0.005), BSP (p<0.05) and osteocalcin (p<0.01) compared to WT and normalized to GAPDH. Bone markers ALP, BMP2 and Runx2 had no significant differences in expression. Hence, the resulting *Rag1^−/−^* primary bone cultures showed a lower capacity for osteoblast differentiation and expression of bone markers compared to WT mice.

Alizarin red staining of colonies (Fig. 4B) from primary mesenchymal stromal cells of *Rag1^−/−^* and WT mice differentiated to osteoblast colony-forming units showed a significant decrease in mineralization at 20 days of culture in *Rag1^−/−^* mice compared to WT suggesting an overall lack of mature osteoblast activity in this T-cell deficient model. There were no differences observed in the confluence or number of colonies between *Rag1^−/−^* and WT mice cultures. Subsequent studies of *Rag1^−/−^* cultures (data not shown) of longer incubation periods greater than 20 days followed by Alizarin red staining showed eventual mineralization similar to that of WT cultures at 20 days previously shown. Thus, mineralization did occur in the *Rag1^−/−^* mice but in a delayed fashion.

Supporting *in vivo* calcein green fluorochrome labeling studies (Fig. 4C) showed continued decreased mineralization and bone formation in *Rag1^−/−^* mice even in the subsequent phases of fracture healing at 14, 21 and 28 day time points compared to WT. Calcein was administered at 2 and 9 days prior to harvest of the limbs. With each fluorochrome label representing the injection time points, the amount of bone formation during this 7 day period was directly reflective of the distance measured between the calcein administrations. Five different representative areas of the endosteum just outside the zone of the fracture callus on 3 different sections were measured per group (n = 3). The measured distances between mineralization fronts were statistically significant at 14 (p<0.05) (image not shown), 21 (p<0.01) and 28 (p<0.01) days. Thus, a consistent overall decrease in osteogenesis was observed in the *Rag1^−/−^* mice throughout the different phases of fracture healing. Unfractured limbs showed no significant differences in bone formation.

### The Fracture Callus from *Rag1^−/−^* Mice Shows Less Healing than Observed in WT Mice

**Figure 5:**
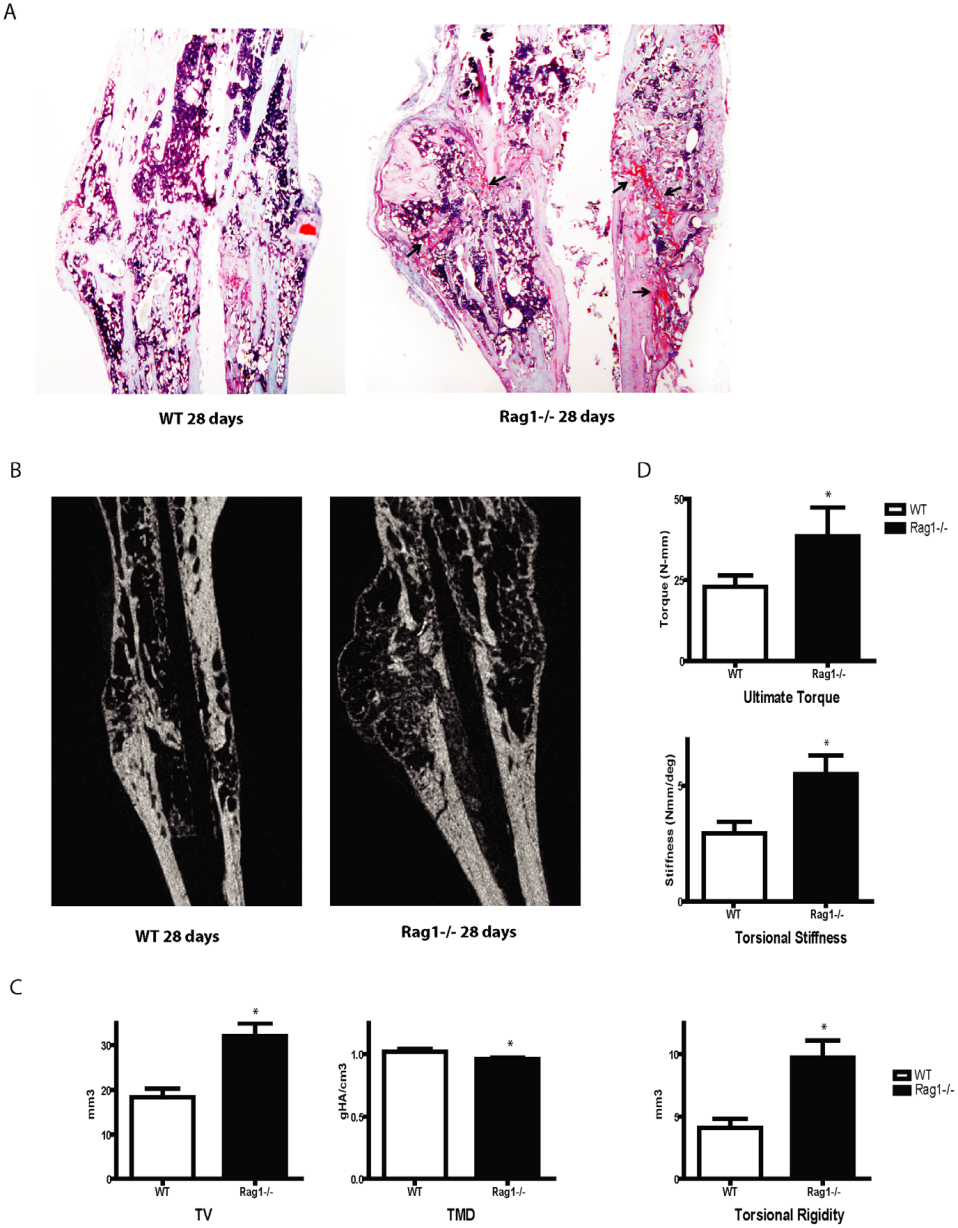
Healing of the fracture callus in *Rag1* *^−^* ^***/****−*^ **mice is delayed.** (**A**) Safranin O staining of WT and *Rag1^−/−^* mice 28 days post fracture showed persistence of a cartilage template with more proteoglycan staining (black arrows) and less bridging bone across the fracture gap in *Rag1^−/−^* compared to WT mice. (**B**) µCT analysis confirmed the presence of a wider, lower density callus in *Rag1^−/−^* mice tibias at 28 days. (**C**) This was reflected by increased total callus volume (TV) measurements (p = 0.002) and decreased tissue mineral density (TMD) at 28 days in the *Rag1^−/−^* compared to WT mice (p = 0.004). Torsional rigidity (TR) was significantly higher in *Rag1^−/−^* mice compared to WT (p = 0.002). (**D**) Mechanical assessment of the samples using torsional mechanical testing showed a significantly higher ultimate torque and torsional stiffness in the *Rag1^−/−^* mice at 28 days compared to WT (p = 0.002).

The differences in callus healing were assessed. Histologic data indicated the persistence of a cartilage template and less endochondral bone formation at 28 days in the *Rag1^−/−^* mice compared to WT mice (Fig. 5A). More proteoglycan staining and less bridging of the fracture gap with bone was apparent in the *Rag1^−/−^* mice. This was further corroborated by longitudinal µCT scan images of the fracture callus which were analyzed using current stereologic/histomorphometric methods (Fig. 5B).

Qualitatively, the results indicated a larger, less healed bridging fracture callus which quantitatively exhibited lower tissue mineral density (TMD, p = 0.004) and higher total callus volume (TV, p = 0.002) in *Rag1^−/−^* mice compared to WT calluses at 28 days post fracture (Fig. 5C). The WT mice demonstrated more advanced remodeling characteristics with a smaller, higher density fracture callus. There were no differences seen (p>0.2) in bone volume fraction (BV/TV), trabecular thickness (Tb.Th), trabecular number (Tb. N.) or trabecular separation (Tb.S.) between groups at 28 days. At 35 days, there were no differences observed between the two groups for any of the parameters (p>0.2). Thus, it appears that bone healing eventually occurs in the *Rag1^−/−^* mice but is delayed in comparison to WT mice.

Biomechanical testing showed higher ultimate torque and torsional stiffness (p = 0.002) in the *Rag1^−/−^* mice at 28 days compared to WT (Fig. 5D). The elevated ultimate torque and torsional stiffness between *Rag1^−/−^* and WT mice was no longer significant at 35 days. Although an unexpected higher torsional stiffness resulted from the biomechanical testing of *Rag1^−/−^* mice, a presumption of better healing cannot be concluded based on this data. The wider distribution of bone deposition around the diaphyseal (neutral) axis, despite its reduced density, resulted in an increased polar moment of inertia, leading to greater torsional rigidity (p = 0.002) (23).

## Discussion

Here we found that a lack of T-cells delayed osteoblast maturation and prolonged the proliferative phase of fracture healing resulting in more immature osteoblasts and decreased bone formation. Quantitative RNA expression studies of the fracture callus and primary bone cultures confirmed the overall decrease in mature osteoblasts correlated with the absence of T-cells and its secreted factors in the early phases of fracture repair. T-cells are selectively recruited in control human fractures during the early phase of fracture repair [26] and T helper cells and macrophages are disproportionately elevated in the fracture site compared to what is seen systemically [4]. This indicates a crucial role for T-cells in osteoblast regulation by subsequently uncoupling the proliferative and remodeling phases of fracture healing such that a lack of T-cells results in delayed and/or impaired bone healing. This is commonly seen clinically in immune deficient conditions such as those related to trauma, autoimmune or malignancy disorders as well as malnutrition and advanced age [14], [27]–[31]. While the factors that determine fracture repair in immune compromised patients are complex, focusing on the T-cell mediated maturation of osteoblasts may provide the critical link to understanding the actual fracture repair mechanism. Our data defines a new role for T-cells in regulating osteoblast differentiation, thus expanding on previous information from investigations studying the role of T-cells in bone of *Rag1^−/−^* [22] and δγT-cell deficient mice [32].

Based on the µCT analysis, the *Rag1^−/−^* mice demonstrated a larger, lower density callus compared to WT at 28 days indicating a more immature state of healing. While this wider but lower density pattern of bone distribution led to an increased polar moment of inertia (PMI) and greater torsional rigidity, this did not indicate a more advanced state of bone healing. Torsional rigidity is particularly relevant to heterogenous materials such as fracture callus/healing bone. Torsional rigidity values calculated will not be equal to the PMI values, but will show the same trends in that a wider cross sectional area will yield both higher PMI and torsional rigidity values. Torsional rigidity, however, is a more appropriate metric to use as it more directly relates to experimentally measured torsional stiffness incorporating both geometric and material parameters vs. PMI which is representative of geometric distribution alone. As such, torsional rigidity was used in this study and has been utilized successfully in the recent biomechanics literature with respect to assessing rodent long bones, including fidelity of healed fractures in rat femora [23], [33].

The cytokine expression data provided support for the concept that an imbalance of pro- and anti-inflammatory cytokines may be the underlying mechanism of osteogenesis. For this reason, we directly treated osteoblast cells in culture with these cytokines to observe their effects on maturation. Indeed, IL-17F alone, promoted increased expression of osteoblast bone markers, highlighting for the first time, a key role for the pro-inflammatory cytokine IL-17F in fracture healing. This suggests a model in fracture healing processes whereby IL-17F, known to be secreted by the T-helper cell 17 (Th17) subset of T-cells, stimulates osteoblast maturation. More importantly, the impaired osteoblast maturation and decreased bone marker expression observed in *Rag1^−/−^* mice primary mesenchymal stromal cells were able to be rescued with direct IL-17F treatment further supporting its osteoinductive role. Immunodeficient *Rag1^−/−^* primary mesenchymal stromal cells demonstrated an impaired osteogenic potential compared to WT mice *in vitro.* The lack of T-cells, including Th17, in the *Rag1^−/−^* mice is an important difference as WT mice have Th17 cells in the bone marrow which have the potential to be stimulated. Although the Th17 cell stimulation was not fracture induced, the addition of osteoblast differentiation media to the cultures after the initial incubation period for cells to adhere did in fact influence bone marker expression in WT cells. As *Rag1^−/−^* mice have an absence of Th17 cells from the outset, the addition of osteoblast differentiation media had little effect in inducing the markers for bone formation. Thus, these results in part support the conclusions that T-cells are important in osteoblast differentiation. However, mesenchymal stromal cells do not express IL-17F and it was also not found to be expressed in osteoblasts differentiated from primary mesenchymal cells *in vitro* (data not shown). Hence, it is postulated that the relevance of a fracture stimulus *in vivo* is key to the up-regulation and expression of this essential pro-inflammatory cytokine to promote bone formation and healing. Factors to which the cells are exposed in the animal *in vivo* before they are harvested will influence cell number or differentiation potential. This is illustrated by data showing that even short term ovariectomy influences osteoblast differentiation when cells are assayed *in vitro* [34].

**Figure 6:**
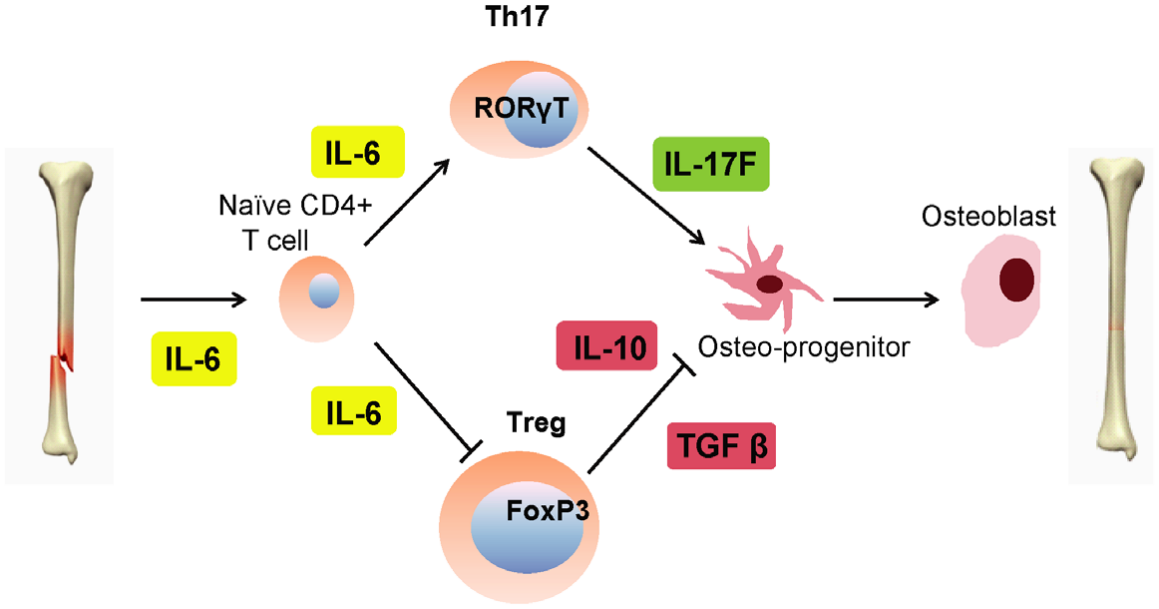
Proposed mechanistic scheme of T-cell mediated osteoblast differentiation and maturation. Upstream IL-6 increase early post fracture promotes naïve CD4+ T-cells to Th17 cells stimulating pre-osteoblast cell differentiation via IL-17F. Concomitantly, the Treg pathway is suppressed, decreasing TGFβ and IL-10 and inhibiting pre-osteoblast differentiation. The effect of IL-6 also has a direct role in the later stages of osteoblast differentiation in fracture healing.

Although IL-6 has modest effects on osteoblast differentiation directly [35], upstream IL-6 increase early post fracture likely promotes naïve CD4+ T-cells into Th17 cells stimulating pre-osteoblast cell differentiation. Activated IL-6 (IL-6 bound to IL-6 receptor (IL-6R)) interacts with gp130, a signal transducing subunit, to phosphorylate Janus kinase (Jak) to activate cytoplasmic transcriptional factors in Th17 cells for the production of IL-17 [14], [36], [37]. Concomitantly, IL-6 has a dual role in the inhibition of the regulatory T-cell (Treg) population [38], [39] which prevents Treg inhibition of osteoblast activity so as to further allow for osteoblast activity to increase beyond its equilibrium state and thus, allowing a net formation of bone to occur during fracture healing (Fig. 6). This is supported by our qPCR expression data from 3 and 7 day fracture callus RNA which revealed the sustained down-regulation of IL-6 in *Rag1^−/−^* mice compared to WT beyond the early phases of healing which resulted in less mature osteoblasts and mineralization.

The decrease in pro-inflammatory cytokines and subsequent impaired fracture healing is substantiated by other studies in the literature. A lack of IL-6 expression in a mouse model was found to have biomechanically weaker calluses during early fracture healing [40]. Furthermore, TNFα, another pro-inflammatory cytokine, was found to facilitate fracture repair, albeit through its actions on the muscle-derived stromal cell population as opposed to direct actions on the osteoblast population [1]. Another study suggests that the pro-inflammatory cytokine IL-1β, accelerates osteoblast differentiation and callus mineralization. However, the authors ultimately did not find any net effects on fracture healing with IL-1β deficiency alone, which they attributed to a multi-factorial contribution by the other pro-inflammatory cytokines [41].

It could be suggested that an alternative explanation to T-cell modulation as the mechanism for osteoblast maturation would be that the *Rag1* gene itself is responsible for the maturation effects on the osteoblast. It is unlikely, however, that expression of the *Rag1* protein in the osteoblast is accountable for these findings. The expression of the *Rag1* gene is very tightly regulated and has no significant activity beyond developing T-cells and B-cells. Moreover, given that the *Rag1* protein is a recombinase and cleaves DNA, its more ubiquitous expression would result in increased tumorigenesis in bone which has not been reported [42].

A proposed mechanism derived from these findings is illustrated in Figure 6. In this model of early fracture repair, the secretion of IL-17F by Th17 T-cells is thought to enable osteoblast maturation and activation, permitting bone synthesis to occur. IL-17F is a more recently recognized addition to the family of pro-inflammatory cytokines [14], [38], [43]. To date, IL-17A and F are known to have roles in immunity augmenting the effects of IL-6 and TNFα, with IL-17F known to be expressed in Th17, natural killer (NK) and γδT-cells [38]. Here, these data suggest a novel link between T-cells and osteoblast biology, with IL-17F being a key element. It is possible that this may be the common regulatory point for bone metabolism as there are studies in literature supporting its role in promoting as well as inhibiting bone formation. One study [44] found that IL-17A suppressed osteoclast differentiation when applied at high concentrations *in* *vitro*, while another report [45] found IL-17 to induce osteoclast formation. Similar to the interaction between the osteoblast and osteoclast as mediated by the RANK-RANKL pathway, whereby secretion of RANKL by osteoblasts and its binding to the RANK receptor on the surface of the osteoclast is required for activation of the osteoclast, IL-17 may have effects on stimulating osteoblasts but at different concentrations, producing either net osteoblast or osteoclast action through its direct actions on the osteoblast. Furthermore, the action of these molecules occurs in the context of a network of other proteins and signaling pathways. It may be that a change in the balance of activity between any of these pathways upstream of IL-17 will produce either net bone formation or resorption.

A different balance of overall pathway activity and IL-17 levels may also explain the differences between the γδT-cell deficient model [32] our results in the *Rag1^−/−^* mice which exhibited improved fracture healing. The *Rag1^−/−^* mouse model used in our study would have produced a more global depletion of lymphocytes, including Th17 cells and therefore, a different cellular and cytokine fracture healing environment. Moreover, γδT-cells have the ability to produce IL-10 and TGFβ certainly suggesting that they have the potential to inhibit osteoblast differentiation [46]. While a recent study [22] also examined fracture repair in *Rag1^−/−^* mice, our study focuses on the mechanism of how the effectors of the adaptive immune system alters mesenchymal progenitor differentiation via an imbalance of pro- and anti-inflammatory cytokines. A decrease in pro-inflammatory cytokines and an increase in anti-inflammatory cytokines in the *Rag1^−/−^* mice, especially IL-10, reported in this study, agree with our results. However, their conclusion of a negative effect of lymphocytes on fracture healing was not reproduced in our study. Nevertheless, the immune system and its cellular activation and expression through cytokines as a mechanism of fracture repair during osteoblast regulation are only now being investigated. Further studies are needed to explore the specific signaling pathways involved in osteoblast differentiation and maturation as modulated by T-cells.

In conclusion, our work has shown that a loss of expression of *Rag1^−/−^*, leading to a global depletion of lymphocytic activity appears to be detrimental to the processes of fracture healing, consistent with the assumption that T-cells are essential in fracture healing. IL-17F not only can stimulate and promote osteoblast maturation, but also has shown to directly rescue impaired healing *in vitro*. In doing so, it may be the key mediator in regulating the balance between a net osteoblast or osteoclast activity. As such, future studies aimed at further elucidating the regulation of IL-17F and its actions on mesenchymal progenitor cells may prove to be pivotal in understanding of the interactions between the immune system and bone healing.

### Significance

This study provides a novel mechanism of interaction between the immune system and bone healing, specifically on how T-cells enable osteoblast maturation via IL-17F. This may provide future molecular targets that may have clinical applications to improve bone healing in those with nonunion or risks of impaired fracture healing.
